# A cross sectional study on factors influencing professionalism in nursing among nurses in Mekelle Public Hospitals, North Ethiopia, 2012

**DOI:** 10.1186/1472-6955-13-10

**Published:** 2014-04-04

**Authors:** Atsede Fantahun, Asrat Demessie, Kahsu Gebrekirstos, Ayalnesh Zemene, Gebre Yetayeh

**Affiliations:** 1 Department of Nursing, College of Health Sciences, Mekelle University, Mek’ele, Ethiopia; 2 Department of Nursing and Midwifery, College of Health Sciences, Addis Ababa University, Addis Ababa, Ethiopia

**Keywords:** Attributes of professionalism, Mekelle public hospital nurses, Nursing, Professionalism

## Abstract

**Background:**

Professionalism is defined as the conceptualization of obligations, attributes, interactions, attitudes, and role behaviors required of professionals in relationship to individual clients and to society as a whole. Professionalism attributes include knowledge, spirit of inquiry, accountability, autonomy, advocacy, innovation and visionary, collaboration and collegiality, and ethics. The study assessed level and attributes of professionalism in nursing in Mekelle, Tigray, Ethiopia.

**Methods:**

Institutional based cross sectional study supplemented by qualitative design was employed. Self administered semi structured questionnaire developed from RANO guideline was used. The FGD guideline was developed from different literatures. Data was analyzed using SPSS 16.0. Descriptive statistics and significance was checked at p < 0.05. Professionalism was measured using ANOVA. Qualitative of data were analyzed using coding technique. Written informed consent was obtained from the nurses and confidentiality was assured for all the information provide.

**Results:**

The mean scores for the nurses in Mekelle public hospitals on the professionalism were 140.50, knowledge (25.06), followed by ethics (25.00). The attitudes of respondents on professionalism were at high, moderate, low and very low level. Pearson product–moment correlation analysis revealed small yet significant associations among several professionalism attributes and characteristics of nurses in Mekelle Public hospitals. Age of respondents and work experience were significantly correlated with total professionalism. Work setting in Mekelle hospital was significantly associated with professionalism. Depending on FGD, the major factors were workload, had no vision, FMOH did not focused nursing as a profession, Weakness of the Ethiopian Nursing Association, lack of life insurance as well as the Health professionals and society’s views of the profession.

**Conclusion:**

Nurses with longer years of experience and the older respondents had significantly related with professionalism. Nurses who join professional organizations had high score on professionalism; and nurses working in Military Hospital had high score of professionalism.

## Background

Nursing is autonomous and collaborative care of individuals of all ages, families, groups and communities, sick or well and in all settings. Nursing includes the promotion of health, prevention of illness, and the care of ill, disabled and dying people [1].

Professionalism is a multi-dimensional concept; there is no one simple, generalizable definition, or how to assess it. We can, however, assess professionalism by considering its individual (attributes, capacities, and behaviours), inter-personal (interactions with other individuals and with contexts) and societal dimensions (social responsibility and morality, political, and economic concerns), and the interactions amongst these dimensions [2].

Professionalism is defined as the conceptualization of obligations, attributes, interactions, attitudes, and role behaviors required of professionals in relationship to individual clients and to society as a whole [3,4].

Nursing professional practice is a commitment to compassion, caring and strong ethical values; continuous development of self and others; accountability and responsibility for insightful practice; demonstrating a spirit of collaboration and flexibility [5]. Nurses who value professionalism exhibited adherence to practice standards and technical (psychomotor) competence [6].

The professional status of nursing often is subjected to both internal and external debate. Historians, sociologists, and nurses themselves struggle to determine whether professionalism is present or absent in the occupation called nursing [7]. For many years, scholars in other fields identified nursing as a semi- profession because of the lack of a university-based education as the entry level, the lack of autonomy, and a paucity of theory and research to serve as a foundation for the field [8].

Study that assessed the levels of professionalism and examined factors associated with professionalism among Korean American registered nurses (RNs) used Hall’s Professionalism Inventory (HPI) scale. Current position in nursing, current employment status, work setting, total years of nursing experience, total years of nursing experience in the United States, location of final degree attainment, and duration of nursing education in the United States were associated with the level of professionalism among Korean American RNs [9].

The Ethiopian Nursing Association (ENA) was formed in 1952 E.C as a member of the health professional council involved in registration &licensing of health professionals in Ethiopia. However, In Ethiopia, there was no published studies describing on professionalism in nursing designed to measure professionalism among nurse’s working in hospitals. The objectives of this study were to assess the factors that influence professionalism in nursing. The outcomes of this assessment would help ENA and FMOH in drafting policies and guiding principles of nursing professionalism in Ethiopia as well as for nurses to confirm their professional status and to examine demographic, experiential, and educational factors associated with professionalism.

## Methods

### Ethical considerations

Permission was obtained from Addis Ababa University College of Health Science School of medicine Department Of Nursing and Midwifery Institutional Review Board (IRB) for approval. Following the approval by IRB, Official letter of co-operation was written to concerned bodies by the Department of nursing and midwifery AAU. Written informed consent was given to nurses and confidentiality assured for all the information provided.

### Design, population, and sample

A cross sectional quantitative study design supplemented by qualitative was employed to measure level of professionalism and examine factors associated with professionalism among nurses working in Mekelle public Hospitals. Simple random sampling was used to select sample of 210 nurses. Data were collected using questionnaire adapted from RANO guideline. The sampling frame consisted of 400 clinical nurses via a self administered questioner. The sample size was calculated using single population proportion formula with confidence level of 95% and 5% significance level. Since the total population is less than 10000, using correction formula the final sample size become 210. Simple random sampling technique was used to select the 210 nurses from the four public hospitals.

### Data collection procedures

The data were collected by self administered structured questionnaire which had two parts. Part I socio demographic variables and part II, the attributes of professionalism in Nursing.

Four focus Group Discussions were conducted among nurses in the four hospitals to help the self administered questionnaire and to provide information on the main factors affecting professionalism. The method for the selection of the nurses for FGD was purposive sampling. In order to get detail information within the group, they were structured by department head and year of working experience: In each FGD the number of participants was 5–10 in number. Each discussion was 30 minutes–1 hour.

### Instrument

The questioner was developed from RNAO guideline, which contains likert scale questions prepared in English. The questionnaire was validated by experts on the profession and pre test was done also to assess the reliability of the questionnaire. The Likert scale responses allow subjects to describe how well their opinions and attitudes about nursing agree with the item statements (5-strongly agree; 1 - strongly disagree). A 34-item scale was used to measure a total score for professionalism and the eight attributes of professionalism in five subscales (5 items for each subscale). Scores for the entire scale may range from 34 to 170, with higher scores indicative of professionalism. In addition to the RANO, questions were asked to elicit demographic characteristics. The demographic questions included age, gender, marital status, religion, and year of nursing experience, professional organization membership, and work setting. FGD was prepared to supplement the quantitative data which was prepared open ended questions.

### Data analysis

#### Data coding, entry, and cleaning

Data were entered into a database using Epi Info and analyzed through the Statistical Package for the Social Sciences software version 16 (SPSS 16). Bivariate analyses and multiple logistic regression models using enter method and leveling professionalism and its attributes using mean and standard deviation, (above mean high and below mean low);and for continuous variables categorical and quarter method was used to examine factors associated with professionalism in nursing. P-value less than .05 was defined as statistically significant.

Descriptive statistics were utilized to describe characteristics of the respondents and to assess the levels of professionalism. Descriptive statistics like frequencies, percentages, means, SDs, and ranges were used to describe study subjects. Inferential analyses including t-tests variance (ANOVA) and Correlation were used to determine differences in professionalism and its attributes, Pearson product–moment correlation to identify factors correlated with professionalism.

The FGD was analyzed manually. First the information was transcribed by arranging notes according to forwarded question. Then the data was grouped based on thematic frame works. The themes were workload, had no vision, FMOH did not focused nursing as a profession, Weakness of the Ethiopian Nursing Association, lack of life insurance as well as the Health professionals and society’s views of the profession.

## Results

### Socio demographic characteristics of respondents

Nearly half, 123(58.57%) were females. The mean age of the respondents was 32.4 years (SD = 8.42) and an average work experience was 10.35 years (SD =9.2) in nursing. With their educational status half of the respondents, 108(51.4%) currently possess BSc degree in Nursing, 101(48.1%) diploma, while only one (0.48%) had masters degree (Table 1).

**Table 1:** Frequency and mean table on socio-demographic characteristics among nurses working in Mekelle zone public hospitals (n = 210), May 2012

**Variable**	**Mean (SD)**	**Frequency**	**Percent**
**Age**	32.40 (8.42)		
**Sex**			
Male		87	41.4
Female		123	58.6
**Religion**			
Orthodox		194	92.4
Muslim		7	3.3
Protestant		6	2.9
Catholic		1	.5
Others		2	1.0
**Ethnicity**			
Tigray		193	91.9
Amara		11	5.2
Oromo		4	1.9
Others		2	1.0
**Educational status**			
Diploma		101	48.1
Degree		108	51.4
Masters		1	.5
**Work experience**	10.35 (9.154)		
Marital status			
Single		82	39.0
Married		119	56.7
Divorced		7	3.3
Widowed		2	1.0
**Membership in professional organization**			
Yes		101	48.1
No		109	51.9
**Work setting**			
Ayder		110	52.4
Mekelle		48	22.9
Quiha		22	10.5
Semen Ez		30	14.3
**Salary**	2168.39 (573.28)		

### Levels of professionalism in nursing and its attributes

The respondents mean score of professionalism was 140.5, ranging from (54–170). Mean scores for the attributes Knowledge (25.06), ethics (25.0), and Advocacy (16.8) were highest, while for the attributes autonomy (11.98), innovation and visionary (11.75) were lowest (Table 2).

**Table 2:** Mean score of professionalism and its attributes in Mekelle zone public hospitals, May, 2012

**Variables**	**Mean**	**SD**
Professionalism score	140.50	19.144
Subscale scores		
Knowledge	25.06	3.923
Ethics	25.00	3.844
Accountability	21.13	3.134
Advocacy	16.79	2.763
Spirit of Inquiry	16.56	2.619
Collaboration and collegiality	12.22	2.096
Autonomy	11.98	2.255
Innovation & visionary	11.75	2.333
Valid N (listwise)	210	

Mean professionalism among groups working in different settings were compared using ANOVA. Accordingly, nurses working in North Command Military hospital showed higher scores (mean = 145.9) than did nurses working in Ayder Referral Hospital (138.9), Quiha (mean = 141.2) and Mekelle hospitals (mean = 140).

Mean scores of professionalism has also shown variation against working experiences. Nurses who worked for 20 or more years had higher scores than those with = <10 years (M = 139.46) and 11–20 years of working experience (Table 3).

**Table 3:** Factors associated with professionalism in nursing among nurses working in Mekelle public hospitals, May, 2012

**Variables**	**Total professionalism mean difference**	**Test values t/F**	**P-values**
Age		1.706	.184
20–29 (n = 97)	137.97		(I & V = .017)
30–39 (n = 63)	141.89		
≥40 (n = 50)	143.66		
Sex		1.717	.050
Male (n = 87)	143.18		(I & V = .006)
Female (n = 123)	138.60		
Marital status		2.326	.100
Single (n = 82)	136.96		
Married (n = 119)	142.74		(I & V = .003, Eth. = .04)
Divorced/widowed (n = 9)	143.11		
Membership in PO			
Yes (n = 101)	141.39	.645	.301
No (n = 109)	139.68		
Study site		1.043	.375
Ayder (n = 110)	138.96		
Quiha (n = 22)	141.18		
Mekelle (n = 48)	140.33		(I & V = .025)
Semeneze (n = 30)	145.90		
Working experience		.941	.392
0–10 (n = 140)	139.46		
11–20 (n = 39)	140.92		(I & V = .053)
≤21 (n = 31)	144.65		
Salary		2.287	.104
≤ 1800 (n = 53)	136.09		
1800–2350 (n = 105)	141.05		(I & V = .047, inq. = .087)
≥2350 (n = 52)	143.88		
Education		.652	.196
Diploma (n = 101)	141.40		
≥degree (n = 109)	139.67		(Acct. = 0.044, know = .029

Note > I & V: Innovation and Visionary; know: knowledge; Acct: accountability; inq: inquiry Eth: ethics.

**Figure 1:**
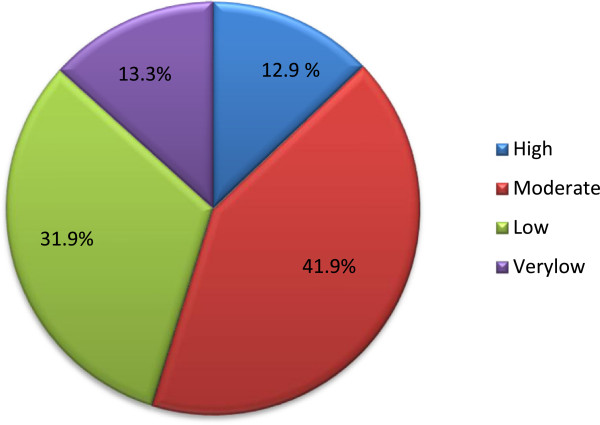
Attitudes of nurses towards professionalism in nursing among nurses working in Mekelle public hospitals May, 2012.

The respondent’s attitude towards concepts of professionalism was calculated using the interval of standard deviation from mean. The attitudes of respondents on professionalism was at high, moderate, low and very low level; 12.9%( n = 27), 41.9% (n = 88), 31.9% (n = 6 7), 13.3% (n = 28) respectively (Figure 1).

### The relationship between socio demographic variables with professionalism and its attributes

Pearson product–moment correlation analysis revealed small yet significant associations among several professionalism attributes and characteristics of nurses in Mekelle Public hospitals (Table 4). The major theme of the FGD was the factors influencing professionalism. It was conducted to compliment to the quantitative findings and to obtain the necessary information to construct the questionnaires and to gain an insider’s perspective of the nurses towards professionalism. The FGD covers a range of questions related to professionalism.

**Table 4:** Correlations between respondent characteristics and professional attributes among nurses in Mekelle public hospitals (n = 210), 2012

**Variables**		**To.prof**	**Knowl.**	**SI**	**Account.**	**Auto.**	**Advoc.**	**I & V**	**CC.**	**Eth.**
Age	r	.122	-.001	.110	.097	.112	.113	.203	.099	.129
	p	.079*	.988	.113	.163	.106	.103	.003**	.152	.063*
Work.exp.	r	.120	.015	.067	.130	.116	.098	.194	.100	.118
	p	.084*	.826	.332	.061*	.093*	.158	.005**	.148	.088*
Salary	r	.095	.052	.105	.038	.065	.117	.130	.056	.085
	p	.170	.449	.130	.585	.350	.091*	.060*	.416	.219

Note = TO = total professionalism; knowl = knowledge; SI = spirit of inquiry; account = accountability; Auto = autonomy; advoc. = advocacy; I & V = innovation and visionary; CC = collaboration and collegiality; Eth = ethics.

**Correlation is significant at the .01 level (2-tailed). *Correlation is significant at the .1 level (2-tailed).

Depending on FGD, the major factors affecting professionalism were workload, lack of vision, lack of support and focus to the profession by government, poor organization of the Ethiopian Nursing Association, lack of life insurance as well as the Health professionals and society’s views of the profession. A diploma male nurse with 20 years of working experience told that “*Remuneration does not compare well with other organizations we learn the same and we are doing the same activity but are paid less.*” and some others said that “*Shortage of staff especially with a huge work load much more than the available staff.*” A diploma female nurse with 15 years of working experience told that “*I don’t think anyone of us will want their child to be a nurse. Except that people do not respect you, it is too much sacrifice for nothing*”*.*

## Discussion

The current study focused on factors influencing professionalism in nursing in Mekelle zone public hospitals. The mean score of professionalism were high compared with findings from Korean American RNs and Wynd 2003. This discrepancy could be due to the sample characteristics. Coming to factors which are associated with professionalism, work setting was important factors affecting professionalism. Similar study from Korean American RNs though several factors such as membership in professional organizations, current nursing employment positions, current employment status, work settings, total years of nursing experience, total years of nursing experience in the United States, location of final degree attainment, and duration of nursing education in the United States were associated with professionalism among Korean American RN. This could be explained that Work setting was only significantly associated with professionalism this may be due to lack of sample size. Nurses who were members of professional organizations, working in Semen Ez military hospital, had many years of experience in nursing practice had a higher total score for total.

Most of the findings were consistent with the study done on 774 American RNs, reported that those nurses who scored highest on levels of professionalism were members of professional organizations, had many years of experience in nursing practice; However, the current study, showed that diploma nurses score high in level of professionalism than degree and Master which is opposite to the above [10]. This may be due to high experience in nursing practice; Study done on 390Army RNs and found that age, length of service, and years of experience were significantly correlated with professionalism [11].This study is similar to the current study found that age and years of experience were significantly correlated with professionalism.

The finding of the study done on perception of 346 nurse’s study in Saudi Arabia showed that nurses had low perception towards their professionalism. Only about one-third of the sample had a high perception of nursing. Low level of perception of professionalism might cause due to these factors: the workplace by itself, personal background of the nurses, which includes the personal interest in the nursing profession, as well as the family’s, society’s and consumer’s views of the profession [12]. The study done in south Africa on 21 nurses managers, the general factors that influenced nurse retention rates were included (1) working conditions and hours, “Hospitals have deteriorated”. This is really not good for patients and the nurses themselves. ‘Working conditions are difficult. With so many patients, some of them very, very ill, nurses feel they are not really giving good quality care. They are despondent and therefore some feel they’d rather leave.’ This finding is similar to the present study. “Nurses in this hospital are working because he/she has no choice.” (2) Professional development opportunities, ‘Training is lacking, especially for the newly qualified nurses, who are not experienced. They do not feel safe, especially with conditions they have never dealt with …’ (3) rewards, ‘Promotion and salary structure must be reviewed. Except that the pay is dreadful, these poor nurses really work hard without considering themselves. Their salaries have got to improve in order to keep them’ and (4) relationships at work, “no respect from the public, families of patients and co-workers.’ ‘Verbal abuse from doctors and some managers must also be stopped, so that nurses remain in their jobs” [13]. The previous studies was similar with the current study which was 12.9 percent only had high professionalism. Moreover, During FGD, low level of perception of professionalism might cause due to the work place, workload, and dissatisfaction with monthly salary and incentives (duty, training etc), had no clear job description, no vision, FMOH did not focused nursing as a profession, Weakness of the ENA, loss of self confidence, poor documentation and reporting, not applying nursing code of ethics, personal interest, knowledge deficit of nursing standards, no enough materials to perform nursing activities, lack of life insurance as well as the HPs and society’s views of the profession.

### Limitations of the study

The factors expected to influence professionalism may not be exhaustive. There could be other influencing factors which our study did not reveal.

More precise measuring instruments, designed to assess actual professional behavioral characteristics, will provide more information to assess behavioral indicators of professionalism along with the attributes of professionalism.

## Conclusion

Nurses with longer years of experience and the older respondents had significantly related with professionalism. Those nurses who join professional organizations had high score on professionalism; and nurses working in Military Hospital had high score of professionalism; however, diploma nurses had high score than degree and above.

Respondents who score moderate attitude toward professionalism were 41.9%. After further statistical analysis using logistic regression, work setting in Mekelle Hospital had significantly associated with professionalism.

On focus group discussion several factors that influence professionalism were raised which was not addressed in the Quantitative data.

The major and common factors in the four governmental hospitals were salary dissatisfaction, low incentives (duty payment, training), lack of refreshment courses to upgrade knowledge, work load, lack of ENA, had no respect with HPs and the society, had no life insurance, FMOH not focus on professionalism in nursing, had clear job description and no vision. This all makes the nurses to have low attitude towards professionalism.

## Abbreviations

AAU: Addis Ababa University; ENA: Ethiopian Nursing Association; EB: Ethiopian Birr; FGD: Focus Group Discussion; FMOH: Federal Minister of Health; HO: Health Officer; HPs: Health Practitioners; HPI: Hall’s Professionalism Inventory; IRB: Institutional Review Board; ICN: International Council of Nurse; NP: Nursing Practitioners; PI: Principal Investigator; RNs: Register Nurses; RNAO: Registered Nurses’ Association of Ontario; US: United States.

## Competing interest

The authors declare that they have no competing interest.

## Authors’ contributions

AF has made substantial contributions to conception and design, or acquisition of data, or analysis and interpretation of data. AD has been involved in drafting the manuscript or revising it critically for important intellectual content. KG has given final approval of the version to be published. AZ has been involving in revising the draft. GY has been contributing in designing and analyzing the data. Finally, all authors read and approved the final manuscript.

## Authors’ information

Atsede Fantahun, Msc in Adult Health Nursing, Lecturer in Mekelle University.

Asrat Demessie, Assisstant Professor and President of Nursing Association.

Kahsu Gebrekirstos, Msc in Child Health Nursing, Lecturer in Mekelle University.

Ayalnesh Zemene, Msc in Maternity and Reproductive Health, Lecturer in Mekelle University.

Gebre Yetayeh, Msc in Adult Health Nursing, Lecturer in Mekelle University.

## Pre-publication history

The pre-publication history for this paper can be accessed here:

http://www.biomedcentral.com/1472-6955/13/10/prepub
